# Bilayer Hydrogel Actuators with High Mechanical Properties and Programmable Actuation via the Synergy of Double-Network and Synchronized Ultraviolet Polymerization Strategies

**DOI:** 10.3390/polym16060840

**Published:** 2024-03-19

**Authors:** Li Tang, Xuemei Wu, Yue Xu, Youwei Li, Shaoji Wu, Liang Gong, Jianxin Tang

**Affiliations:** 1 College of Life Sciences and Chemistry, Hunan University of Technology, Zhuzhou 412007, China; tangli_352@163.com (L.T.); gl569940808@126.com (L.G.); 2 School of Materials Science and Engineering, South China University of Technology, Guangzhou 510641, China

**Keywords:** bilayer hydrogels, hydrogel actuators, mechanical properties, UCST, hydrogen bond, smart materials

## Abstract

Bilayer hydrogel actuators, consisting of an actuating layer and a functional layer, show broad applications in areas such as soft robotics, artificial muscles, drug delivery and tissue engineering due to their inherent flexibility and responses to stimuli. However, to achieve the compatibility of good stimulus responses and high mechanical properties of bilayer hydrogel actuators is still a challenge. Herein, based on the double-network strategy and using the synchronous ultraviolet (UV) polymerization method, an upper critical solution temperature (UCST)-type bilayer hydrogel actuator was prepared, which consisted of a poly(acrylamide-co-acrylic acid)[MC] actuating layer and an agar/poly(N-hydroxyethyl acrylamide-co-methacrylic acid)[AHA] functional layer. The results showed that the tensile stress/strain of the bilayer hydrogel actuator was 1161.21 KPa/222.07%. In addition, the UCST of bilayer hydrogels was ~35 °C, allowing the bilayer hydrogel actuator to be curled into an “◎” shape, which could be unfolded when the temperature was 65 °C, but not at a temperature of 5 °C. Furthermore, hydrogel actuators of three different shapes were designed, namely “butterfly”, “cross” and “circle”, all of which demonstrated good actuating performances, showing the programmable potential of bilayer hydrogels. Overall, the bilayer hydrogels prepared using double-network and synchronous UV polymerization strategies realized the combination of high mechanical properties with an efficient temperature actuation, which provides a new method for the development of bilayer hydrogel actuators.

## 1. Introduction

Hydrogels are soft polymeric materials with a three-dimensional network structure formed through the chemical or physical crosslinking of monomers/polymers [1,2,3,4,5]. By introducing stimulus-responsive monomers into a hydrogel network, hydrogels can respond to external stimuli (light, temperature, pH, etc.) [6,7,8]. Therefore, stimulus-responsive hydrogels show great potential for applications in the field of actuators [9,10,11,12,13]. Bilayer hydrogels, which are the most widely studied hydrogel actuators, consist of a stimulus-responsive actuator layer and another functional layer. When stimulated externally, the actuator-layer hydrogel of the bilayer actuator undergoes a volume change, while the functional layer does not or undergoes an opposite volume change, which allows the bilayer hydrogel actuator to bend, thus producing more complex deformations [14,15,16,17]. However, most bilayer hydrogel actuators have a low driving efficiency and poor overall mechanical properties.

To improve the driving efficiency and mechanical properties of hydrogel actuators, various methods have been reported, such as introducing physical interactions [18], constructing double-network structures [19], adding nanofillers [20,21,22] and utilizing high osmotic pressures to increase the stress [23]. Dai et al. [24] proposed the use of nanofillers to enhance hydrogels for the first time to improve the mechanical properties of hydrogels using cellulose CNC nanocrystals oxidized by a weak oxidizing agent, TEMPO, as nanofillers for carboxymethyl cellulose (CMC)/hydroxyethyl cellulose (HEC) hydrogels, and a comparative analysis of hydrogels reinforced by traditional nano-ceramic materials showed that with CNC nanofillers as the filling, the mechanical properties of pure hydrogels were substantially improved [25]. Gonzalez et al. [26] proposed that the addition of CNC nanofillers to poly(vinyl alcohol) (PVA) hydrogels prepared with a high tensile strength, elongation at break and transparency had a wide range of applications in the field of artificial tissues [27]. However, by constructing a double-network structure or modifying it though adding nanofillers, the driving properties were not improved despite the enhanced mechanical properties. Xu et al. proposed inducing the formation of heterogeneous structures by applying texturized air-cloth face paper [28]. This hydrogel actuator was rapidly and bi-directionally actuated to achieve fast bending (140.6° s^−1^ within the first 5 s) and bending amplitude (850.0° within 30 s) in hot water, as well as fast recovery in cold water, but its mechanical properties were poor.

Herein, we designed a high-strength UCST-type MC-AHA bilayer hydrogel driver by combining the synchronized UV polymerization strategy with the double-network method [29]. Compared to single-layer MC hydrogels (tensile stress and strain: 1205.94 KPa and 231.88%; compressive stress at MC hydrogel strain: 683.81 KPa) and AHA hydrogels (tensile stress and strain: 1188.96 KPa and 232.57%; compressive stress at AHA strain: 896.14 KPa), the MC-AHA bilayer hydrogel had a tensile stress and strain of 1161.21 KPa and 222.07% and a compressive stress of 759.87 KPa at the MC-AHA strain. In addition, MC-AHA bilayer hydrogel actuators maintain excellent stability of pattern programming by curling at low temperatures (5 °C) and stretching at high temperature (65 °C), realizing the precise matching between the passive layer and the driver layer. In this work, the compatibility of high mechanical properties and excellent driving performance of hydrogel actuators was achieved, meanwhile broadening the applications of double-layer hydrogel actuators.

## 2. Materials and Methods

### 2.1. Materials

Acrylamide (AAM, ≥99%), agar (Agar), methacrylic acid (MMA, >99%) and photo initiator (I184, ≥98%) were purchased from Shanghai Aladdin Bio-Chem Technology Co., Ltd. (Shanghai, China). Acrylic acid (AAC, >99%) was purchased from Shanghai Macklin Biochemical Technology Co., Ltd. (Shanghai, China). N-hydroxyethyl acrylamide (HEAA, 98%) was purchased from TCI (Shanghai) Development Co., Ltd. (Shanghai, China). N,N-methylene bisacrylamide (MBA, 98%) was purchased from J&K Scientific Ltd. (Beijing, China); deionized water used in this study was purified via a water purification system.

### 2.2. Preparation of the Hydrogel Actuator

#### 2.2.1. Preparation of the MC Hydrogel Actuator

AAM, AAC, MBA, I184 and H_2_O were weighed (the addition of MBA and I184 was 1 mol% of the addition of AAM and AAC) and dissolved at room temperature with stirring at 500 rpm for 30 min to form a pre-polymerization solution of MC hydrogels. The pre-polymerization solution was injected into a silica gel mold with a syringe, and the MC temperature-sensitive hydrogels were prepared by UV polymerization at 20 °C for 10 min.

#### 2.2.2. Preparation of the AHA Hydrogel Actuator

Agar, HEAA, MBA, I184 and H_2_O were weighed and dissolved by stirring at 95 °C for 30 min, and then MMA was added and stirred uniformly to form an AHA hydrogel pre-polymerization solution, in which the amount of MBA and I184 added was 1 mol% of the amount of HEAA and MMA added. The pre-polymerization solution was injected into the silica gel mold with a syringe while it was hot and then left to cool for 15 min at room temperature. After the first network of agar was formed, it was polymerized under UV for 10 min to form the second network of P(HEAA-co-MMA), and then the AHA hydrogel was prepared.

#### 2.2.3. Preparation of the MC-AHA Bilayer Hydrogel Actuator

MC-AHA bilayer hydrogels were prepared by synchronous UV polymerization. Firstly, AHA hydrogel precursor solution A and MC hydrogel precursor solution B were prepared, and then precursor solution A was poured into the mold and cooled at room temperature to form the first network of agar; after cooling, precursor solution B was injected into the upper gap of the mold and then left to stand at room temperature for 15 min; then precursor solution B was placed under synchronous polymerization under the ultraviolet lamp for 20 min to prepare MC-AHA bilayer hydrogels with the temperature-responsive behavior of UCST. AHA bilayer hydrogels with UCST-type temperature-response behavior were prepared.

### 2.3. Scanning Electron Microscopy Characterization

MC-AHA bilayer hydrogels were freeze-dried after brittle breakage in liquid nitrogen and bilayer hydrogel morphology, by Zeiss Sigma 300 (Oberkochen, Germany).

### 2.4. Fourier Transform Infrared (FTIR) Spectroscopy Tests

Fourier transform infrared spectrometer (Thermo Scientific Nicolet iS5, Waltham, MA, USA) was used to characterize the MC hydrogels and AHA hydrogels using a KBr tablet method.

### 2.5. Mechanical Property Tests

#### 2.5.1. Tensile Property Tests

The tensile property tests of the MC, AHA and MC-AHA bilayer hydrogel actuators were carried out by using a universal tester (AGS-X-10KN, SHIMADZU, Suzhou, China). The size of the sample was a dumbbell shape with a gauge length of 17 mm, a width of 4 mm and a thickness of 4 mm. The tensile speed of the test was 100 mm/min. After the tensile test, the tensile stress–strain curve of the hydrogel actuator was obtained, and the integral area and a slope of 5–10% strain of curves were defined as the bulk toughness and elastic modulus of samples, respectively.

#### 2.5.2. Compression Property Tests

The compression tests of the MC, AHA and MC-AHA bilayer hydrogel actuators were carried out by using a universal tester (AGS-X-10KN, SHIMADZU, Suzhou, China). The size of the sample was a cylindrical shape with a diameter of 15 mm and a height of 20 mm. The compressed speed of the test was 5 mm/min. At the compression test, the compression stress–strain curve of the hydrogel actuator was obtained, and the integral area and a slope of 0–10% strain of curves were defined as the bulk toughness and elastic modulus of samples, respectively.

#### 2.5.3. Use of 90° Peeling Tests

The interfacial toughness of the MC-AHA bilayer hydrogel and MC+AHA hydrogel (MC and AHA hydrogels were synthesized separately, and then MC and AHA were pressed together for 1 h) actuators was determined by using a 90° peeling test. The peeling speed of the test was 100 mm/min. Interface toughness was defined as the maximum failure load that the interface could bear per unit width (that is, the maximum failure energy that could be endured per unit area), which was estimated by interface toughness = F_max_/W, where F_max_ is the 90° peeling test during peak force and W is the width of the sample.

#### 2.5.4. Fatigue-Resistance Tests

A fatigue-resistance test of the AHA hydrogel actuator was carried out by using a universal tester (AGS-X-10KN, SHIMADZU, Suzhou, China). The size of the sample was a dumbbell and cylindrical shape that was tensile and compressed to 30% and 50% strains at 100 mm/min and 5 mm/min and then unloaded to 0% strain at the same tensile rate for 10 consecutive loading–unloading cycles without any intervals. At the end of the successive loading–unloading experiments, the loading–unloading curves of the AHA hydrogels were obtained, and the integral area of the curves was defined as the dissipated energy (U_hys_), which was calculated by the following formula: U_hys_ = $\int_{\varepsilon =0}^{\varepsilon =\varepsilon_{x}}\left(\sigma_{load}-\sigma_{unload}\right)d\varepsilon$; where ε_x_ is the preset strain, and σ_load_ and σ_unload_ are the corresponding stresses in the loading and unloading processes, respectively.

### 2.6. Deswelling Tests

The UCST of MC hydrogels was obtained by deswelling tests. Taking 20 mm × 20 mm × 2 mm MC hydrogels as the sample, the mass (g) of the sample, which was incubated for 3 h under different temperatures (5–95 °C), was measured, and then the samples were dried at 60 °C and weighed to obtain the initial mass (g_0_), and the sample swelling rate (g/g_0_) was calculated.

### 2.7. Actuating Performance Tests

MC-AHA hydrogel actuators of 50 mm × 5 mm × 2 mm were used as samples, and the bending angles of the samples were measured in an aqueous solution at temperatures of 65 °C and 5 °C. Furthermore, three different shapes of hydrogel actuators were designed, “butterfly”, “cross” and “circle”.

## 3. Results and Discussion

### 3.1. Fabrication of the MC-AHA Bilayer Hydrogel Actuator

**Figure 1:**
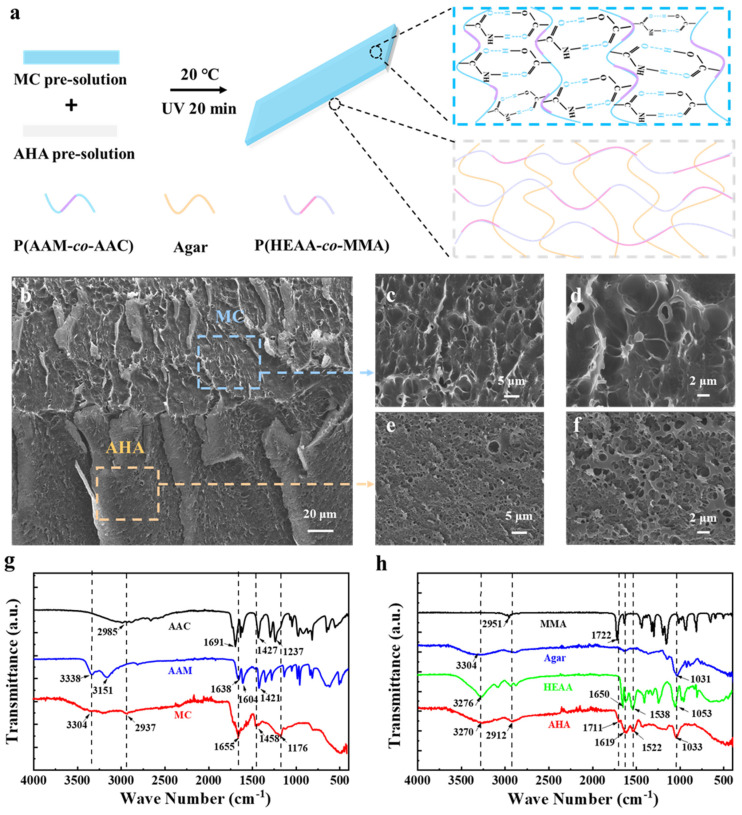
(**a**) Preparation schematic and (**b**–**f**) SEM characterizations of MC-AHA bilayer hydrogels. (**g**) Fourier transform infrared spectroscopy of MC hydrogels. (**h**) Fourier transform infrared spectroscopy of AHA hydrogels.

As shown in Figure 1a, an MC-AHA bilayer hydrogel actuator is prepared using a synchronous UV polymerization method. Specifically, agar, HEAA, MBA, I184 and H_2_O were selected to construct an AHA pre-solution. When the pre-solution was heated-cooled, the agar in it was transformed from the sol state to the gel state, and then a flat single network hydrogel was formed in the mold. Next, AAM, AAC, MBA, I184 and H_2_O were mixed to obtain an MC layer pre-solution, which was poured onto the AHA layer and allowed to gradually permeate into it though molecular diffusion. Finally, the mold was photopolymerized under 365 nm UV for 30 min. During the photopolymerization process, HEAA monomers in the AHA layer formed a flexible polymer network, constructing a double-network structure with a rigid agar network to provide strong mechanical support for the bilayer hydrogel actuator. AAM in the MC layer copolymerized with AAC to form a temperature-sensitive polymer network to endow the bilayer hydrogel with an actuating ability. In the interfacial region between the AHA and MC layers, monomers of the MC layer permeated into the AHA layer, and then the permeated monomers copolymerized with the monomers of the AHA layer, forming strong covalent bindings. Meanwhile, the newly generated copolymer chains were interspersed in the original network to construct a topological entanglement structure, thus achieving high interfacial toughness in the bilayer hydrogel actuator. Therefore, the bilayer hydrogel actuator prepared based on UV polymerization possessed strong mechanical properties, high interfacial toughness and good actuating ability.

The morphology of the MC-AHA bilayer hydrogels was characterized using SEM. Figure 1b shows a cross-section of the morphology of MC-AHA hydrogels. Obviously, hydrogel actuators have a tightly adherent bilayer structure, which consists of an MC hydrogel driving layer and an AHA double-network hydrogel passive layer (Figure 1c–f).

The multiple dynamic interactions within the MC and AHA hydrogel network were verified through FTIR characterization. As shown in Figure 1g, the FTIR spectrum confirms the formation of a co-polymer of acrylamide and acrylic acid, as is evident from bands that appeared in the range of 2937 cm^−1^ to 3304 cm^−1^ (O–H and N–H stretching) in the MC hydrogels. In addition, the characteristic peaks of C=O stretching vibration in MC hydrogels shifted from 1691 cm^−1^ and 1638 cm^−1^ to 1655 cm^−1^ compared to AAC and AAM, respectively, indicating that the main mode of interaction between AAC and AAM is hydrogen-bonding interaction.

In Figure 1h, MMA shows an O–H stretching signature at 2951 cm^−1^ and a C=O telescoping vibration signature at 1720 cm^−1^. Agar shows an O–H telescoping vibration signature at 3440 cm^−1^. HEAA shows distinct signature peaks at 1650 cm^−1^ (C=O telescoping-vibration signature peak), 1538 cm^−1^ (N–H bending-vibration signature peak), 1053 cm^−1^ (C–O stretching-vibration characteristic peak) and 3276 cm^−1^ (hydrogen active-wave characteristic peak). Meanwhile, all the characteristic peaks of MMA, agar and HEAA were observed in the AHA hydrogel without new peaks. In addition, the active hydrogen peaks, C=O stretching-vibration peaks and N–H bending-vibration peaks of AHA hydrogels shifted from 3276 cm^−1^, 1650 cm^−1^ and 1538 cm^−1^ to 3270 cm^−1^, 1619 cm^−1^ and 1522 cm^−1^ compared with those of HEAA, which suggests that the main interaction mode of MMA, agar and HEAA is hydrogen bonding. Therefore, the crosslinking mode of AHA hydrogels is attributed to the all-physical crosslinking that was dominated by hydrogen bonding.

### 3.2. Mechanical Properties of the MC-AHA Bilayer Hydrogel Actuator

**Figure 2:**
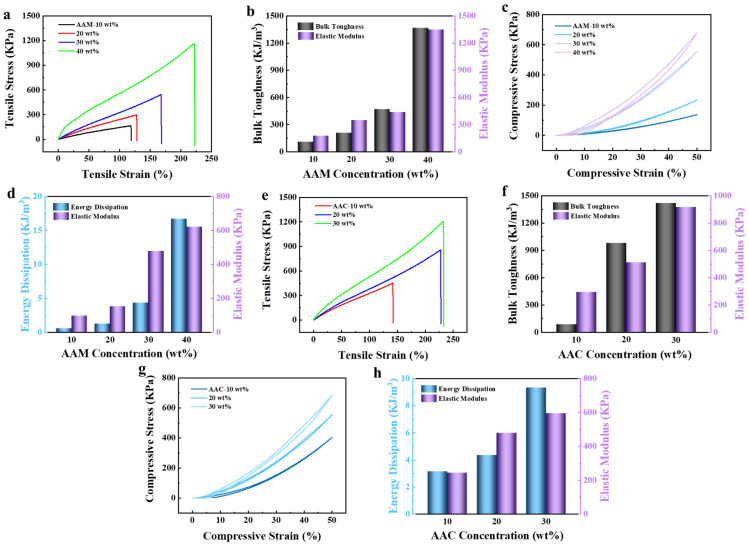
MC hydrogels with different AAM concentrations: (**a**) tensile stress–strain curve, (**b**) toughness and elastic modulus, (**c**) compressive stress–strain curves and (**d**) energy dissipation and elastic modulus. MC hydrogels with different AAC concentrations: (**e**) tensile stress–strain curve, (**f**) toughness and elastic modulus, (**g**) compressive stress–strain curves and (**h**) energy dissipation and elastic modulus.

The effects of different concentrations of AAM on the mechanical properties of MC hydrogels were investigated. As shown in Figure 2, the mechanical properties of MC hydrogels increased with the increase in monomer concentrations. When the concentration of AAM was increased from 10 wt% to 40 wt%, the tensile stress/strain of MC hydrogels increased from 166.44 KPa/118.54% to 1163.02 KPa/221.58%, and the toughness/modulus of elasticity increased from 108.27 KJ/m^3^/173.71 KPa to 1365.79 KJ/m^3^/1349.90 KPa (Figure 2a,b). This was attributed to the increased concentration of AAM monomers, which resulted in the formation of more hydrogen bonds and denser network crosslinking in the gels, thus enhancing the mechanical properties of the MC hydrogels. As the concentration of AAM increased, the compressive stress of MC hydrogels increased from 136.83 KPa to 679.18 KPa, and the dissipation energy/elastic modulus increased from 0.58 KJ/m^3^/97.90 KPa to 16.73 KJ/m^3^/620.57 Kpa (Figure 2c,d). This could be attributed to the large number of hydrogen bonds in the gel network, which were able to dissipate more energy.

Considering that hydrogels need to maintain good flexibility during actuation to avoid adverse effects on the actuation performance of bilayer hydrogel actuators due to too much stiffness, we chose an AAM concentration of 30 wt% to continue studying the effects of AAC at different concentrations on the mechanical properties of MC hydrogels. When the AAC concentration increased from 10 wt% to 30 wt%, the tensile stress/tensile strain increased from 451.97 KPa/141.88% to 1205.94 KPa/231.88%, and the toughness/modulus of elasticity increased from 83.51 KJ/m^3^/293.48 KPa to 1418.91 KJ/m^3^/917.91 Kpa (Figure 2e,f), respectively. The compression performance is shown in Figure 2g,h. As the concentration of AAC monomers increased, the compression stress/elastic modulus/dissipation energy increased from 402.88 KPa/243.45 KPa/3.14 KJ/m^3^ to 683.81 KPa/595.55 KPa/9.32 KJ/m^3^.

**Figure 3:**
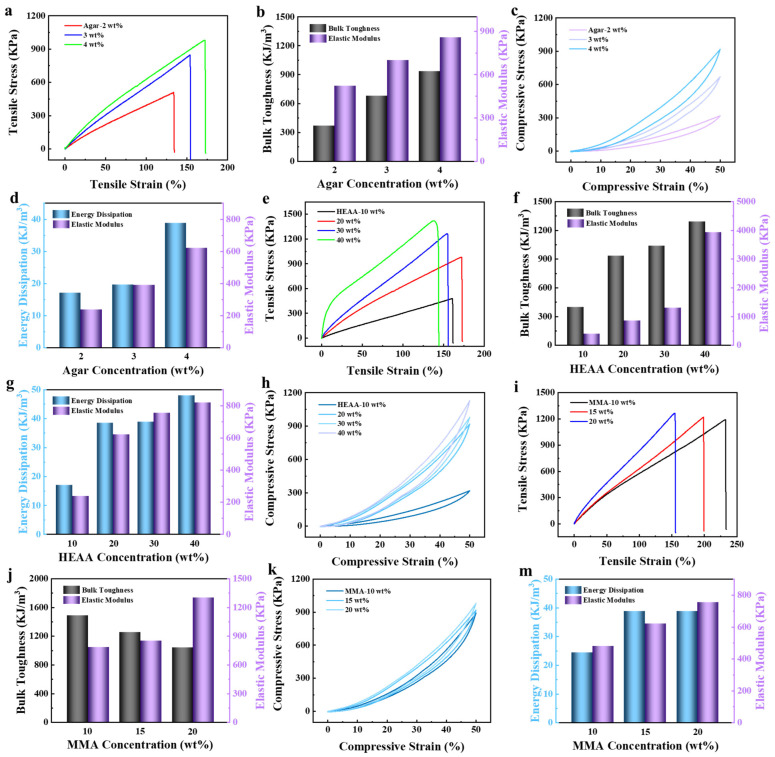
AHA hydrogels with different agar concentrations: (**a**) tensile stress–strain curve, (**b**) toughness and elastic modulus, (**c**) compressive stress–strain curves and (**d**) energy dissipation and elastic modulus. AHA hydrogels with different HEAA concentrations: (**e**) tensile stress–strain curve, (**f**) toughness and elastic modulus, (**g**) compressive stress–strain curves and (**h**) energy dissipation and elastic modulus. AHA hydrogels with different MMA concentrations: (**i**) tensile stress–strain curve, (**j**) toughness and elastic modulus, (**k**) compressive stress–strain curves and (**m**) energy dissipation and elastic modulus.

The mechanical properties of AHA hydrogels are mainly related to the crosslinking of the monomers, so the effects of components of different concentrations on AHA hydrogels were investigated separately. Firstly, the effects of different agar concentrations on the hydrogels were investigated. With an increase in agar concentration from 2 wt% to 4 wt%, the tensile stress/strain of the hydrogels increased from 506.69 KPa/133.84% to 979.50 KPa/171.98%, and the corresponding elastic modulus/toughness increased from 521.04 KPa/366.42 KJ/m^3^ to 858.31 KPa/934.06 KJ/m^3^, respectively (Figure 3a,b). This was because agar acted as a rigid network in AHA double-network hydrogels, and the higher the monomer concentration was, the more hydrogen bonds would be formed. Therefore, the high-density hydrogen-bond crosslinking network of hydrogels is beneficial to improving their mechanical properties. At the same time, the compressive stress/elastic modulus/dissipated energy also increased from 318.53 KPa/236.59 KPa/17.01 KJ/m^3^ to 918.45 KPa/621.80 KPa/38.79 KJ/m^3^, which was consistent with the tensile analysis.

The effects of HEAA composition on the mechanical properties of AHA hydrogels are shown in Figure 3e–h. When the concentration of HEAA was increased from 10 wt% to 40 wt%, the tensile stress/modulus of elasticity/toughness of the hydrogels increased from 474.75 KPa/394.38 Kpa/398.44 KJ/m^3^ to 1419.14 Kpa/3929.93 Kpa/1290.83 KJ/m^3^, respectively, where the maximum tensile strain of 171.98% was found at an HEAA concentration of 20 wt%. However, the tensile strain showed a trend that first increased and then decreased, which exhibited a completely different trend from the others. This indicated that with the increase in HEAA concentration, more HEAA polymer chains were formed in the hydrogel network, which formed more interchain entanglement and caused the hydrogels to change from an elastic state to plastic; therefore, their stretching was reduced. Figure 3g,h show the variations in the compressive properties of AHA hydrogels with HEAA concentrations, which were consistent with the tensile properties. The compressive stress increased from 318.53 KPa to 1129.78 KPa, and the corresponding elastic modulus/dissipated energy increased from 236.59 KPa/17.01 KJ/m^3^ to 819.97 KPa/47.97 KJ/m^3^.

When the concentration of MMA increased from 10 wt% to 20 wt%, the tensile stress/elastic modulus of AHA hydrogels increased from 1188.96 KPa/786.28 KPa to 1263.79 KPa/1301.08 KPa, while their tensile strain/toughness decreased from 232.57%/1487.73 KJ/m^3^ to 154.72%/1039.90 KJ/m^3^ (Figure 3i,j), and their compression stress/elastic modulus/dissipation energy increased from 896.14 KPa/479.13 KPa/24.43 KJ/m^3^ to 978.14 KPa/754.58 KPa/38.79 KJ/m^3^ (Figure 3k,m), respectively. Because of the increase in concentration, the crosslink density of the second network, which had stronger dissipation energy when subjected to external forces, was greater.

Through the optimization of each of the above components, we finally selected 30 wt% AAM and 30 wt% AAC as fixed ratios of the MC actuator layer, with 4 wt% agar, 30 wt% HEAA and 10 wt% MMA as the fixed ratios of the AHA function layer for the construction of the bilayer hydrogels.

**Figure 4:**
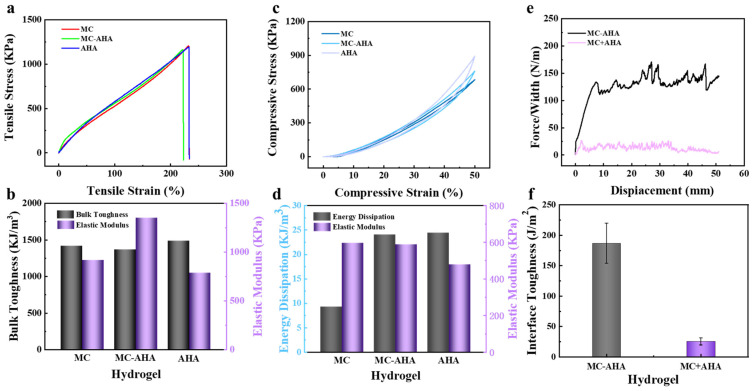
MC, AHA and MC-AHA hydrogels: (**a**) tensile stress–strain curves, (**b**) toughness and elastic modulus, (**c**) compressive stress–strain curves and (**d**) energy dissipation and elastic modulus. Interfacial toughness of MC-AHA bilayer hydrogels. (**e**) Typical peeling-force/width curves of MC-AHA bilayer hydrogels and MC+AHA bilayer hydrogels at a peeling rate of 100 mm/min. (**f**) Comparison of the interfacial toughness of MC-AHA bilayer hydrogels and MC+AHA bilayer hydrogels.

As shown in Figure 4, the mechanical properties of the three hydrogels were similar, and the MC-AHA bilayer hydrogels had the combined mechanical characteristics of MC and AHA hydrogels. The tensile stress/strain measurements of the MC hydrogels, AHA hydrogels and MC-AHA bilayer hydrogels were 1205.94 KPa/231.88%, 1188.96 KPa/232.57% and 1161.21 KPa/222.57%, respectively. The elastic modulus/toughness measurements of the MC and AHA hydrogels were 917.91 KPa/1418.19 KJ/m^3^ and 786.28 KPa/1487.74 KJ/m^3^, while the toughness and elastic modulus of the MC-AHA bilayer hydrogels were 1365.79 KJ/m^3^ and 1349.90 KPa, respectively (Figure 4a,b). Since the MC-AHA bilayer hydrogels were a combination of MC and AHA hydrogels, their modulus of elasticity was larger than that of single-layer hydrogels, and bilayer hydrogels are harder as a whole. In addition, the compression performance of the double-layer hydrogels also reflected the combination of bilayer hydrogels. Figure 4c,d show the compression performance of three kinds of hydrogels, and the compression stresses of MC, AHA and MC-AHA hydrogels were 683.81 KPa, 896.14 KPa and 759.87 KPa, respectively. The corresponding dissipated energy and moduli of elasticity were 595.55 KPa and 9.32 KJ/m^3^, 479.13 KPa and 24.43 KJ/m^3^, and 589.01 KPa and 24.05 KJ/m^3^, respectively.

High interfacial toughness can prevent interlayer separation of the bilayer hydrogel actuator, which is the key to the stable application of the bilayer hydrogel actuator. Therefore, the interlayer adhesive properties of the MC-AHA hydrogel actuators were studied by 90° peeling tests. Firstly, MC-AHA hydrogels were synthesized though synchronous UV polymerization in order to investigate the effect of a synchronous UV polymerization on the interfacial toughness of the bilayer hydrogel actuator. As shown in Figure 4e,f, the interfacial toughness of the MC-AHA bilayer hydrogel actuator synthesized though synchronous UV polymerization was better than that of the MC+AHA bilayer hydrogels, which increased from 25.41 ± 11.87 J/m^2^ to 186.59 ± 58.41 J/m^2^. Clearly, synchronous UV polymerization had a great effect on the interfacial toughness of the bilayer hydrogel actuator. This is attributed to the infiltration of monomers from the upper layer into the lower layer prior to polymerization and the formation of an interlocking polymer topological network between the two layers after UV polymerization, thus improving the interfacial toughness.

### 3.3. Temperature-Sensitive Behavior and Actuating Mechanism of the MC-AHA Bilayer Hydrogel Actuator

**Figure 5:**
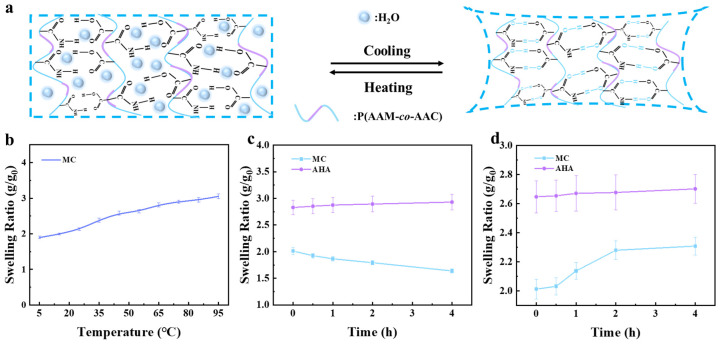
(**a**) Mechanism of UCST phase transition in MC hydrogels. (**b**) Swelling curve of MC hydrogels. Swelling ratio of MC and AHA hydrogels as a function of time at (**c**) 5 °C and (**d**) 65 °C.

The temperature sensitivity of MC hydrogels depends on the association and dissociation of hydrogen bonds among the MC polymer chains. As shown in Figure 5a, an MC network formed through AAM and AAC copolymerization contains a large number of intermolecular hydrogen bonds. When the ambient temperature is lower than the UCST, the amide groups and carboxyl groups polymerize to form a large number of intermolecular hydrogen bonds, which form a “zipper-like” structure among the chains, and the intermolecular chains become more densely interconnected, the water molecules are excluded and the gel volume shrinks. On the contrary, when the ambient temperature is higher than its UCST, intermolecular hydrogen bonding dissociates and combines with water molecules, the polymer chains are loosened and the gels absorb water and expand. Therefore, the UCST of MC hydrogels was measured through a swelling experiment according to the differences in the water content of MC hydrogels at different temperatures.

As shown in Figure 5b, the swelling curves indicate that the swelling rate of MC hydrogels increased gradually with the increase in external temperature, showing UCST-type temperature-responsive behavior, which is mainly attributed to the gradual dissociation of intermolecular hydrogen bonds. The most significant changes in the swelling volume of MC hydrogels were observed above and below 35 °C, so the UCST of MC hydrogels was determined to be about 35 °C. As shown in Figure 5c,d, MC hydrogels showed different swelling behaviors with temperature changes, with the swelling rate decreasing at 5 °C and increasing at 65 °C, which is consistent with the UCST-type temperature-responsive behavior of MC hydrogels. In addition, the swelling trend of AHA hydrogels at ambient temperatures of 5 °C and 65 °C was synchronized, not accompanied by temperature changes, and the swelling rate increased slowly with increasing time with no obvious temperature-responsive behavior.

**Figure 6:**
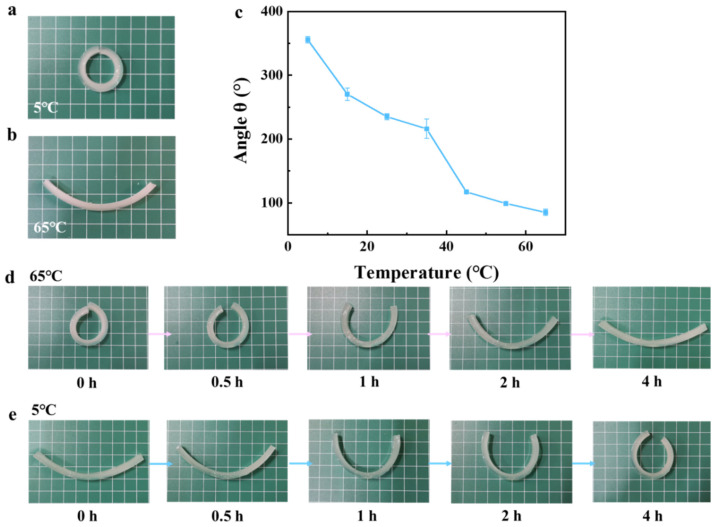
Diagram of MC-AHA bilayer hydrogels actuating at (**a**) 5 °C and (**b**) 65 °C. (**c**) Driving angles at different temperatures. Actuation performance of the MC-AHA bilayer hydrogel actuator as a function of time at (**d**) 65 °C and (**e**) 5 °C.

Based on the UCST-type temperature-responsive behavior of MC hydrogels, we investigated the temperature-dependent actuation of MC-AHA bilayer hydrogels. As shown in Figure 6a,b, the MC-AHA bilayer hydrogel actuator was able to fully curl into a “◎” shape at an ambient temperature of 5 °C, while the “◎” stretched into an arcuate curve at an ambient temperature of 65 °C. This may be due to the fact that AHA hydrogels are not temperature-sensitive, and when the hydrophilic monomers in the hydrogels are sufficiently bound to the water molecules, the hydrogels swell with water absorption and cannot be reversibly absorbed/dehydrated with temperature changes. Figure 6c shows the actuation of the MC-AHA bilayer hydrogel actuator in the temperature range of 5–65 °C. The bending angle (θ) of the MC-AHA bilayer hydrogel actuator decreased as the ambient temperature increased, with the most significant change in the bending of the hydrogels being from 235° to 117° above and below the UCST of MC hydrogel (~35 °C). This could be attributed to the UCST-type temperature-responsive behavior of MC hydrogels. When the ambient temperature exceeds the UCST, a large number of intermolecular hydrogen bonds are dissociated, and a large number of ambient water molecules rush into the interior of the hydrogels to combine with amide and carboxyl groups, resulting in an expansion of the volume of MC hydrogels.

In addition, the driving ability of the MC-AHA bilayer hydrogel actuator was further investigated at ambient temperatures of 5 °C and 65 °C. As shown in Figure 6d, an MC-AHA bilayer hydrogel actuator of an “◎” type was placed at 65 °C, and with the increase in time, the “◎”-type hydrogels gradually stretched and stabilized after 4 h and finally stretched into a curved curve (the bending angle was 59°). The MC-AHA bilayer hydrogel actuator was subjected to an ambient temperature of 5 °C, and the bilayer hydrogels started to curl again with time, with a maximum bending angle of 286° after 4 h (Figure 6e). This might be due to the fact that the hydrophilic monomers of AHA hydrogels absorbed water, leading to the expansion of the gel volume, and when the ambient temperature was changed, it could not reversibly absorb/lose water and reached an equilibrium of swelling over a long period of time in an aqueous environment, so that when the temperature was lowered, the MC-AHA bilayer hydrogel actuator could not be completely curled into the original “◎” shape, which is in line with the above analysis. Therefore, the swelling of MC and AHA hydrogels at different temperatures was investigated separately.

### 3.4. Actuating Performances of the MC-AHA Bilayer Hydrogel Actuator

**Figure 7:**
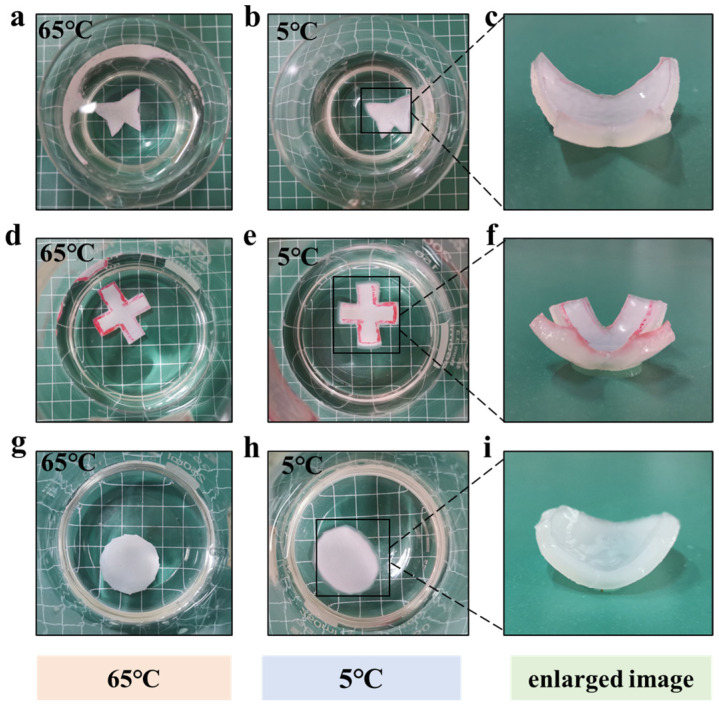
Drive shape changes in MC-AHA bilayer hydrogels at 65 °C and 5 °C: (**a**–**c**) butterflies, (**d**–**f**) crosses, (**g**–**i**) circle.

The MC-AHA bilayer hydrogels were used as the research object, and three types of hydrogel actuators were constructed based on shape design. As shown in Figure 7, the “butterfly”, “cross” and “circle” did not bend at 65 °C, and they bent at 5 °C, and the degree of bending was not much affected by the shape change. This shows that MC-AHA bilayer hydrogels have great potential for programmability and can be adapted to different applications, which achieves the goal of high-strength bilayer hydrogel actuators.

## 4. Conclusions

In short, a high-strength UCST-type MC-AHA bilayer hydrogel driver was prepared using the synchronous UV polymerization strategy and double-network design. The high-strength AHA bilayer hydrogels had a tensile stress and strain of 1161.21 KPa, 222.07%, as well as a compressive stress of 759.87 KPa. Meanwhile, due to the existence of a large number of intermolecular hydrogen bonds in the MC, the “zipper-like” hydrogen bonds gradually dissociated/conjugated when the temperature of the external environment changed, and it had the ability to demonstrate UCST-type temperature response. Therefore, the MC-AHA bilayer hydrogel actuator had different volume differences with temperature changes, which could be curled into a “◎” shape at 5 °C, and the “◎” could be stretched out at 65 °C so as to realize good actuation without being affected by the shape of the hydrogel. It had excellent programmability and drive stability, showing great potential in the field of hydrogel actuators.

## Data Availability

The raw data supporting the conclusions of this article will be made available by the authors on request.
